# Supplementary figures and images for: Response of Cucumis sativus to Neighbors in a Species-Specific Manner
Source: Plants (Basel). 2022 Dec 27;12(1):139. doi: 10.3390/plants12010139 (PMC9824612; doi:10.3390/plants12010139)

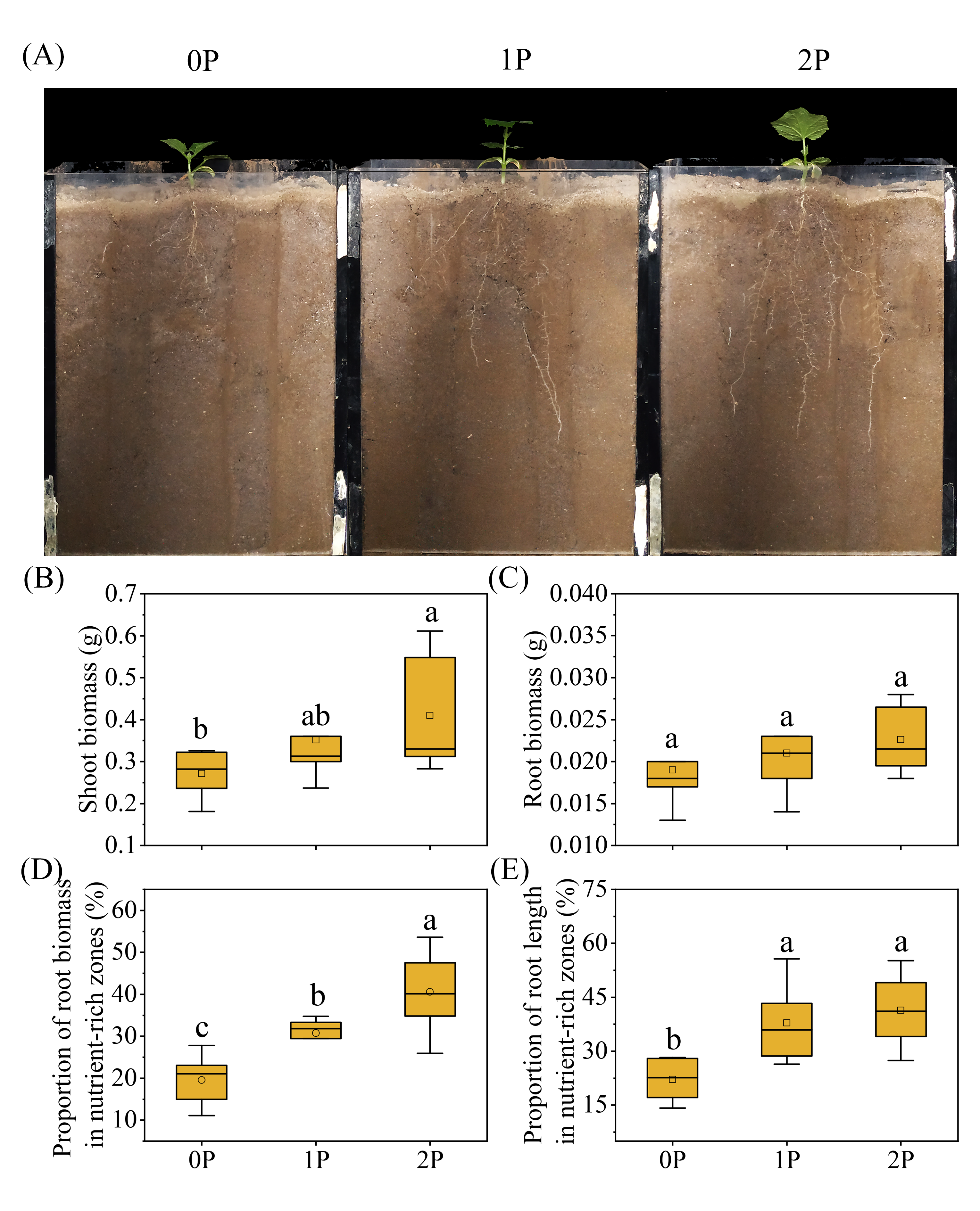

Supplement: Supplementary file 1 [file plants-12-00139-s001.zip › Figure S1.tif]
